# The prevalence of mental health problems among users of NHS stop smoking services: effects of implementing a routine screening procedure

**DOI:** 10.1186/1472-6963-11-190

**Published:** 2011-08-16

**Authors:** Lisa McNally, Chloe Todd, Elena Ratschen

**Affiliations:** 1 Public Health Department, Surrey NHS Primary Care Trust, Cedar Court, Leatherhead, Surrey, KT22 9AE, UK; 2 UK Centre for Tobacco Control Studies, University of Nottingham, Clinical Sciences Building, City Hospital, Nottingham, NG5 1PB, UK

## Abstract

**Background:**

Tobacco dependence among people with mental health problems is an issue that deserves attention both from a clinical and from a public health perspective. Research suggests that Stop Smoking Services often fail to ask clients about underlying mental health problems and thus fail to put in place the treatment adaptations and liaison procedures often required to meet the needs of clients with a mental health condition who want to stop smoking. This study assesses the recording of mental health problems in a large NHS stop smoking service in England and examines the effect of implementing a short screening procedure on recording mental health conditions.

**Methods:**

Treatment records from the Stop Smoking Service covering a period of 13 months were audited. The prevalence of reported mental health problems in the six month period before the implementation of the mental health screening procedure was compared with that of the six month period following implementation. The screening procedure was only implemented in the support services directly provided by the Stop Smoking Service. Comparisons were also made with third-party sections of the service where no such screening procedure was introduced.

**Results:**

The prevalence of reported mental health problems among a total of n = 4999 clients rose from less than 1% before implementation of the screening procedure to nearly 12% in the period following implementation, with the change being statistically significant. No significant rise was observed over the same period in the sections of the service where no screening procedure was implemented.

**Conclusions:**

The absence of standard procedures to record mental health problems among service users in many stop smoking services is currently likely to prevent the detection of co morbidity. Implementing a simple screening procedure appears suitable to increase the routine recording of mental health problems in a stop smoking service, which is an essential step to ensure services can be tailored and delivered appropriately to the client group.

## Background

There have been a number of calls for smoking cessation support to be made more accessible and appropriately tailored to those living with mental health conditions [1,2]. These calls have been based on the very high levels of smoking in this group and the resulting, well-documented consequences in relation to both physical [3] and mental well being [2], which in conjunction contribute considerably to the health inequalities experienced by this population.

Review level evidence suggests that levels of motivation to quit smoking among people with mental health conditions can be comparable to that found within the general population [4]. Complex psychosocial and neurobiological factors associated with the link between smoking and mental illness however pose challenges to addressing the issue in this group. Smoking, for example, still constitutes the norm rather than the exception in many mental health settings, and while it is 'de-normalised' in the rest of society, the smoking culture in mental health settings appears to be prevailing. Important neurobiological links refer to metabolic interactions between psychotropic medication and constituents in tobacco smoke, which require higher doses of medication for smokers to achieve therapeutic blood levels, and consequently necessitate a review and potential decrease of dosage following cessation [2]. Awareness that withdrawal from nicotine may pose a challenge to mental well-being [5] and can, if untreated, mimic or exacerbate symptoms of mental illness^1^, is important, and enhanced mood or stress management intervention may need to be incorporated into quit support. Appropriately tailored support and liaison with all treatment providers are therefore paramount to ensure that smoking treatment can be safe and successful [6].

Recent research suggests that the majority of NHS Stop Smoking Services do not proactively screen for mental health problems or collect data on the proportion of their clients that have mental health issues [7]. Therefore, it may be that these services are regularly supporting smokers with mental health conditions to quit without knowledge of those problems and without making the appropriate adjustments to their treatment programme.

The AIMS procedure [7] has been proposed to enable efficient screening and recording of mental health status for NHS Stop Smoking Services. AIMS is an acronym for *Ask, Inform, Medication *&*Support*. It is intended to ensure that Stop Smoking Service *ask *all smokers requesting support to quit about their mental health, *inform *the client's mental healthcare provider about the quit attempt, ensure the issue of *medication *metabolism is addressed and then tailor the stop smoking *support *to the specific mental health needs of the client (eg: flexible sessions, enhanced psychological support).

The aim of this study is to review NHS Stop Smoking Service data in order to assess the prevalence of recorded mental health conditions on the database of a large NHS Stop Smoking Service, and to examine changes in recording prevalence before and after implementing the AIMS screening procedure.

## Methods

The study data were taken from an audit of a large English NHS Stop Smoking Service (Surrey) covering a total population of over 1 million people and a period of 13 months (from May 2009 to May 2010). The treatment provided by the Stop Smoking Service falls into two main categories: Direct services are those provided directly by the Stop Smoking Service team within specialist (group-based) clinics or via telephone support. In contrast, Third-Party services are commissioned by the Stop Smoking Service and are provided by suitably trained professionals in General Practice or Community Pharmacy settings.

Six months into the data collection period, the AIMS screening procedure was implemented within the directly provided services. Hence from this point, all clients using directly provided services were routinely being asked whether or not they have any mental health problems (as well as about the nature of any current treatment if applicable). Screening for mental health problems took place at the first point of contact between the client and the Stop Smoking Service.

The 'inclusion' criteria for recording were based purely on clients' self report and their definition of symptoms as representing a mental health problem or condition. Mental health problems were recorded even if there had been no formal diagnosis or treatment received. Stop Smoking Service staff did not have access to the clients' medical records or any other sources of data that may have provided information on clients' mental health history.

No screening procedure was implemented across the Third Party services. The professionals commissioned to provide these services were not informed of the screening procedure implementation within the direct services.

The recorded prevalence of mental heath conditions across the six-month period before the implementation of the screening procedure (Period 1 - May 09 to October 09) was compared with the recorded prevalence across the six-month period after implementation (Period 2 - December 09 to May 10) using Chi-Squared tests. Separate analyses were carried out for both Direct and Third Party services. The month in which the AIMS Procedure was first implemented into the direct services (November 09) was excluded from all analyses so as to allow for a transition period during which staff within the direct services became familiar with using the AIMS screening procedure.

## Results

**Figure 1:**
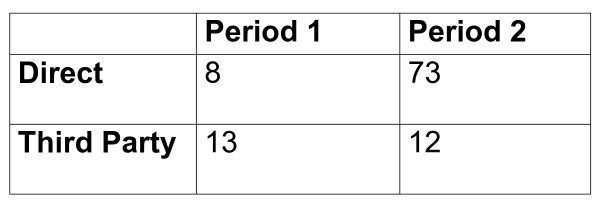
**Distribution of clients recorded as having a mental health problem by service type and study period (n = 106)**.

A total number of n = 4999 clients were recorded as having set a quit date in the study period (once November was excluded). Among this sample, 51% were female and the average age was 44 years (std = 14.64). A total of 1667 quitters used treatment provided with the Direct stop smoking services and 3332 quitters used services provided by the Third-Party providers in General Practice or Community Pharmacy. In total, 106 quitters reported mental health problems (see Figure 1)

**Figure 2:**
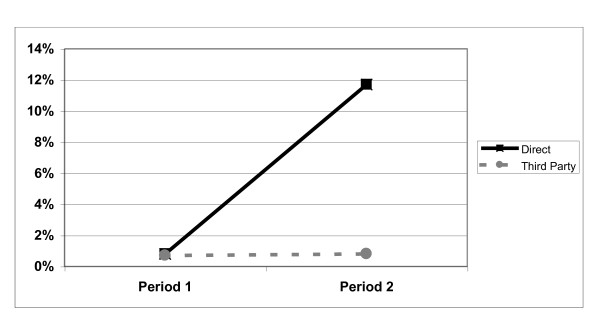
**Prevalence of reported mental health problems by service type and study period**.

Across all services, the recorded prevalence of current mental health problems among clients was significantly higher in Period 2 (4.1%) than it had been in Period 1 (0.7%) (Chi^2 ^= 67.68, p < 0.001). Examining third party services alone (where AIMS had not been implemented), there was no significant increase in the prevalence of mental health problems (0.7% to 0.8%: Chi^2 ^= 0.24, ns). However, there was a significant increase among clients using direct services (where AIMS had been implemented) (0.8% to 11.7%: Chi^2 ^= 100.34, p < 0.001) (see Figure 2).

Among the 106 clients reporting current mental health problems, only 2 were not also currently engaged in treatment for these problems. Among clients receiving mental health treatment, 27 (25%) were in receipt of specialist care (defined as having a care coordinator or psychiatrist); the remaining clients were receiving mental healthcare delivered within General Practice.

## Discussion

In the absence of routine and proactive screening for mental health conditions, the recorded prevalence of such conditions among clients setting a date to quit smoking was below 1% across both directly provided and Third-Party provided stop smoking services. Following the introduction of routine screening for mental health conditions within direct services, the recorded prevalence rose to nearly 12% (more than 1 client in every 9). Except in two cases, clients reporting mental health problems were also currently receiving mental health treatment, with a quarter of these under the care of specialist mental health services.

Limitations of this study include the fact that there will inevitably have been uncontrolled variance in how clients defined a 'current mental health problem'. It seems clear that, at least in this study, clients were normally only reporting such problems if they were receiving treatment for them. The 'true' prevalence of mental health problems among the sample, including those undiagnosed, is therefore likely to have been underestimated. According to the Adult Psychiatric Morbidity Survey (APMS), as many as 23% of the population are affected by a mental health condition, if mild and moderate conditions are included [8].

It is also notable that the location for this study (South East England) is likely to have a lower than average prevalence of mental health problems among its residents. For example, the APMS data suggests approximately 10% of men in the South East have a common mental health problem compared to 15% in the North West [8]. Following this ratio, the mental health prevalence of 12% found among Stop Smoking Service Clients in Surrey may therefore appear as approximately 18% in a North West service.

Overall, this study suggests that NHS Stop Smoking Services are currently likely to treat a significant number of clients each year while being unaware that they have current mental health problems. This may be a particular issue when clients access quit support outside of primary care settings and where medical records may not be readily available (recent data suggests that 55% of quit attempts in England occur outside of primary care settings) [9]. The adoption of a simple screening procedure such as AIMS into service routines is feasible and can significantly increase the extent to which service providers are aware of client's mental health problems.

The findings of this study have clear implications for practice within NHS Stop Smoking Services. As mentioned earlier, when supporting a client with a mental health condition to stop smoking, it is essential to put in place appropriately tailored support in relation to medication and/or psychological well-being [7]. The present study suggests that at least 1 in 9 clients of NHS Stop Smoking Services will have a current mental health condition and require such treatment adaptations. However, in the absence of routine mental health screening, NHS Stop Smoking Service staff will often remain unaware that a mental health condition exists and such adaptations will therefore not occur. Given previous evidence that the majority of NHS Stop Smoking Services do not operate mental health screening procedures or treatment protocols [7] there is an urgent need for a widespread change in practice.

## Conclusions

A significant number of people approaching NHS Stop Smoking Services may also happen to be living with mental health conditions. If these services are to offer a consistently safe and effective intervention for all of its clients, then routine screening of all clients for mental health problems should be undertaken.

## Competing interests

LM has previously received funding for her work from pharmaceutical companies.

## Authors' contributions

LM designed the study, screening procedures and led on the writing of the manuscript. ER and CT both contributed to the analysis and interpretation of data, as well as drafting and revising the manuscript. All authors have given final approval of the version published.

## Pre-publication history

The pre-publication history for this paper can be accessed here:

http://www.biomedcentral.com/1472-6963/11/190/prepub
